# Induction treatments with and without addition of one dose anthracycline to all-trans retinoid acid and arsenic in pediatric non-high-risk acute promyelocytic leukemia: study protocol for a randomized controlled trial

**DOI:** 10.1186/s13063-024-08664-y

**Published:** 2024-12-18

**Authors:** Zhong Fan, Xiu-Ya Huang, Dan-Ping Huang, Jie-Si Luo, Jia-Yin Su, Xiao-Li Zhang, Yu Li, Li-Na Wang, Cong Liang, Xue-Qun Luo, Li-Bin Huang, Yan-Lai Tang

**Affiliations:** Department of Pediatrics, The First Affiliated Hospital, Sun Yat-Sen University, Guangzhou, China

**Keywords:** Acute promyelocytic leukemia, Children, Arsenic trioxide, Realgar-Indigo naturalis formula, Chemotherapy

## Abstract

**Background:**

The treatment of all-trans retinoic acid (ATRA) and arsenical agent has revolutionarily improved the prognosis of acute promyelocytic leukemia (APL) both in adults and children. Nevertheless, coagulation disorder and differentiation syndrome (DS) are the main causes of early death in APL patients. Early chemotherapy to reduce leukocytes during induction is an important measure to reduce complications and mortality. However, the incidence of hyperleukocytosis (WBC > 10 × 10^9^/L) was significantly higher in pediatric patients without chemotherapy than in adults. Although ATRA plus arsenic is the standard therapy for non-high-risk adult patients, it remains controversial whether chemotherapy is necessary for induction therapy in pediatric APL.

**Methods:**

This study was designed as a multicenter randomized controlled trial. Children with APL were randomly assigned into experimental group (ATRA-RIF plus chemotherapy) and control group (ATRA-RIF). The experimental group was treated with ATRA-RIF plus chemotherapy for induction, while the control group was treated with ATRA-RIF alone. In addition, both groups received the same regimen of ATRA-RIF plus chemotherapy for consolidation and maintenance.

**Discussion:**

This trial aims to compare the efficacy of ATRA-RIF plus chemotherapy versus ATRA-RIF in pediatric non-high-risk patients with APL to demonstrate that chemotherapy during induction therapy can reduce the incidence of complications such as hyperleukocytosis and DS, thereby reducing mortality.

**Trial registration:**

Chinese Clinical Trials Registry, ID: ChiCTR2000038877. Registered on October 8, 2020, https://www.chictr.org.cn/showproj.html?proj=60733. V1.0 date 08/01/2020.

**Supplementary Information:**

The online version contains supplementary material available at 10.1186/s13063-024-08664-y.

## Background

Therapy with arsenic and all-trans retinoic acid (ATRA) substantially improves the long-term survival rate of acute promyelocytic leukemia (APL). Two arsenic compounds, intravenous arsenic trioxide (ATO) and Realgar-Indigo naturalis formula (RIF) which is an oral traditional Chinese medicine mainly containing Realgar (As_4_S_4_), have been clinically used in treatment of the disease. Randomized studies showed that treatment efficacy is comparable between patients treated with ATO and RIF, including adult and pediatric patients [1–4].

It has been reported that non-high risk (NHR) APL (initial white blood cell count (WBC) ≤ 10 × 10^9^/L) in adults was successfully treated by induction therapy with a chemotherapy-free combination of ATRA and arsenic [5–8]. However, this needs to be verified in pediatric patients. There is concern that the use of the two differentiating agents without chemotherapy may result in an increasing risk of leukocytosis which is an important factor in the development of differentiation syndrome (DS) [9, 10]. DS is one of the major causes of early death and DS-related death rate can be as high as 5.7% [11]. Previous studies suggested that the incidence of leukocytosis (> 10 × 10^9^/L) in pediatric patients with NHR APL was 84–100% and much higher than 35–47% in adult counterpart, if treated with chemotherapy-free induction [6, 7, 12–14]. In fact, when comparing two prospective clinical trials conducted in children with APL [1, 3], it is found that the incidence of DS is much higher in patients on induction with chemotherapy-free combination of ATRA and arsenic than in those receiving induction with one dose anthracycline and the chemotherapy-free combination. The total incidence of DS including NHR and high-risk patients (initial white blood cell count > 10 × 10^9^/L) was 41% in the former and 6% in the latter. Furthermore, small sample retrospective studies showed that in children with NHR APL received chemotherapy-free induction with ATRA-ATO combination, most of them developed leukocytosis [15, 16]. These patients require discontinuation of ATO and ATRA and/or hydroxyurea and cytarabine therapy to reduce leukocytes. Therefore, the chemotherapy-free induction therapy should be evaluated further in the treatment of children with APL in randomized clinical trial, although this induction therapy has been successful used in adult patients.

Hemorrhage is another main cause of early death in APL and mainly occurs before hematological complete remission (HCR) of the disease [17, 18]. The rate of hemorrhagic death during induction period remains at about 5–10% in the clinical trial setting [11]. Coagulopathy is triggered by APL blasts. ATRA and arsenic are known to improve the biological signs of APL coagulopathy. However, patients are still at risk of fatal hemorrhage due to coagulopathy until complete HCR of APL is achieved. Circumstantial evidence suggests that median time to achieve HCR of APL may be shorter in pediatric patients receiving additional one dose of anthracycline to the chemotherapy-free combination in induction therapy (24 days) than in those receiving the chemotherapy-free combination only (32 days) as well as the hospital days required for inpatient management during induction [1, 3].

Therefore, South China Children Cancer Group (SCCCG) conducted a prospective, randomized, controlled, multicenter superiority study to compare the efficacy and safety between induction treatments with and without addition of one dose anthracycline to all-trans retinoid acid and arsenic in pediatric NHL APL. The ultimate aim of the study is to determine the safety and feasibility of chemotherapy-free induction therapy, which has been successful used in adult patients, in the treatment of pediatric NHR APL.

## Methods

### Study hypothesis

It is known that there are important distinctions between pediatric and adult patients with APL [19]. Based on the circumstantial evidence, we hypothesize that induction therapy with additional one dose of anthracycline to ATRA-arsenic combination can reduce the incidence of leukocytosis and DS, the time for achieving HCR, and hospital days required for inpatient management in pediatric patients with NHR APL.

### Eligibility criteria

Patients will be recruited from 29 hospitals in South China enrolled in SCCCG-APL study. Information of the hospitals enrolled are listed in Supplementary Table 1. All study participants will receive detail information about the study and the treatments and sign informed consent before participation. The participants will be able to withdraw from the study at any time without giving reasons.

#### Inclusion criteria

Participants fulfilling the following:

1. Age ≤ 16 years.

2. Newly diagnosed APL with PML-RARα/t (15; 17).

3. Initial peripheral blood WBC ≤ 10 × 10^9^/L (NHR APL).

#### Exclusion criteria

Participants meeting one or more of the following:

1. Death from any cause before genetic diagnosis and randomization
2. Coma, convulsion, paralysis due to intracranial hemorrhage, cerebral thrombosis, or central nervous system leukemia before genetic diagnosis and randomization
3. Prolonged QT syndrome at diagnosis
4. Declined randomization

### Sample size calculation

Based on the previous studies [3] assuming 80% hyperleukocytosis rate in the control (ATRA-RIF) group and conservatively assuming 45% hyperleukocytosis rate in the experimental (ATRA-RIF plus chemotherapy) group, 2.5% type I error (one-sided), and 80% power, we used PASS 11 to calculate a required sample size of 30 evaluable patients per group to detect a between-group difference. Allowing for a withdrawal rate of 5% would require 32 patients per group. Therefore, we plan to enroll 64 patients into two groups (1:1).

### Randomization

**Fig. 1:**
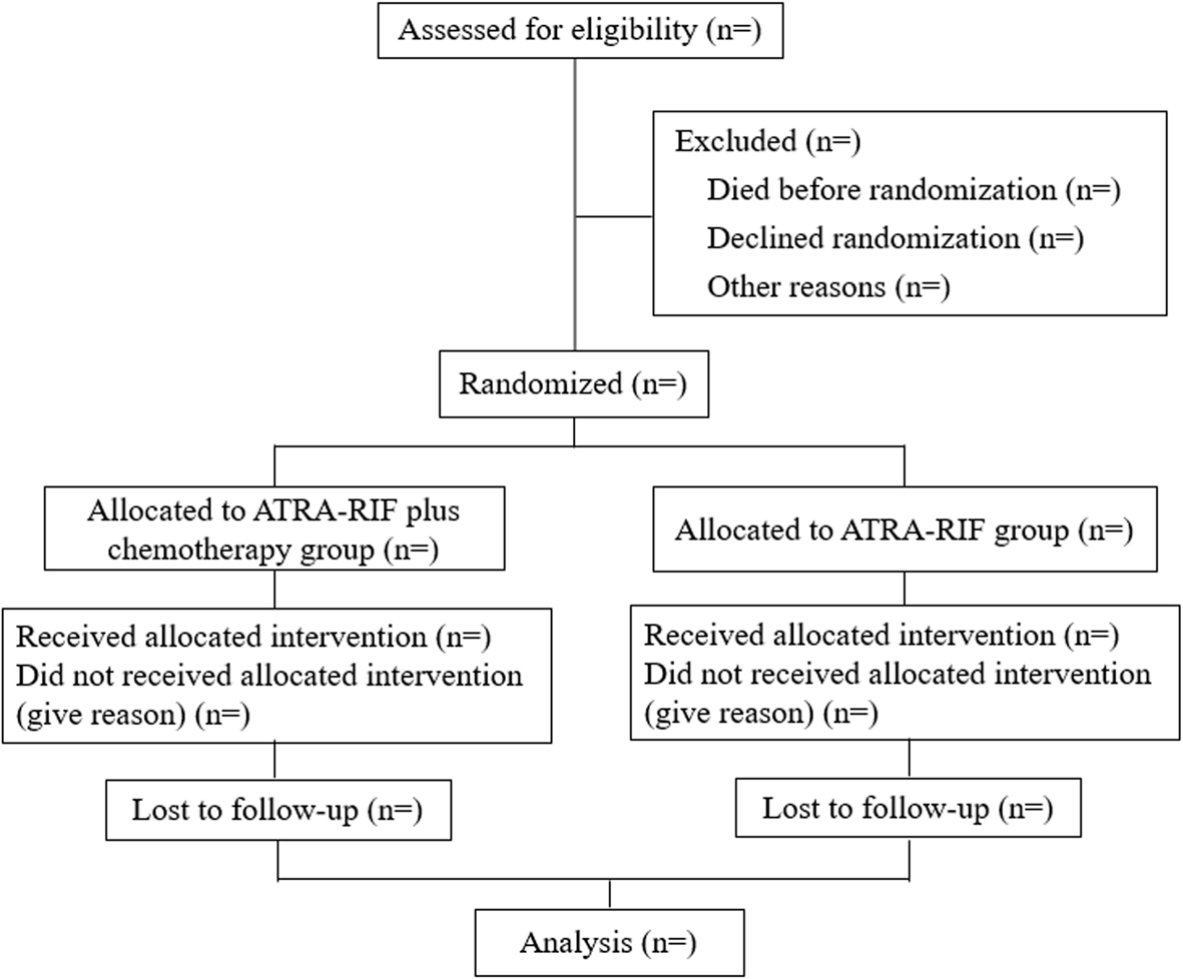
Trial flowchart

Once informed consent is obtained, participants will be randomly assigned (1:1) by a computer-generated, random-allocation schedule to one of two groups: the ATRA-RIF plus chemotherapy group (experimental group) and ATRA-RIF group (control group) (Fig. 1). A clinical research coordinator, operating independently, will allocate patients to either group. The creation of the allocation sequence for participants will be overseen by a second researcher.

### Blinding

Blinding method will not be used in this study because it is not feasible for this study for patients and the research team. Both patients and researchers are informed of the grouping.

### Interventions

**Fig. 2:**
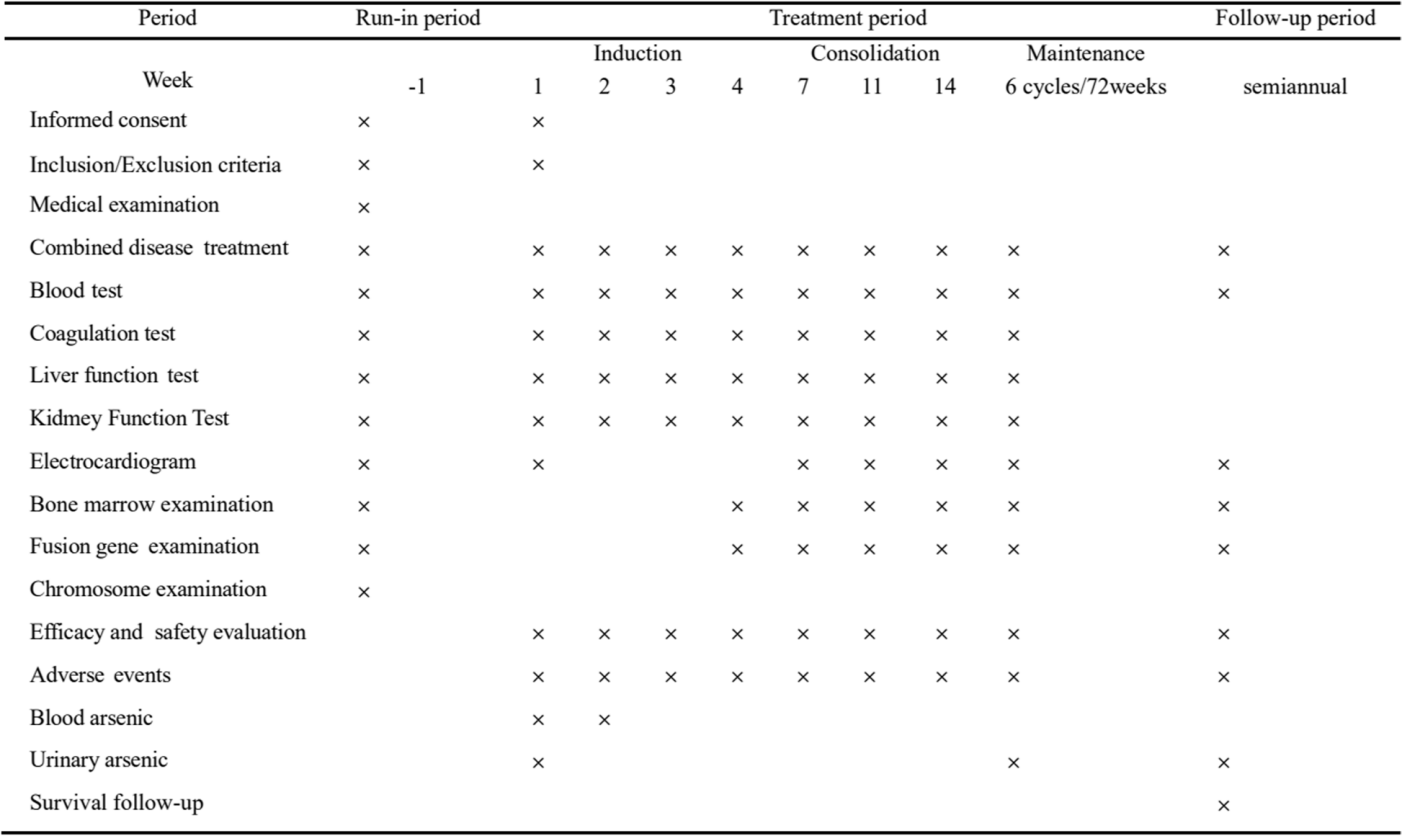
Trial process chart

**Fig. 3:**
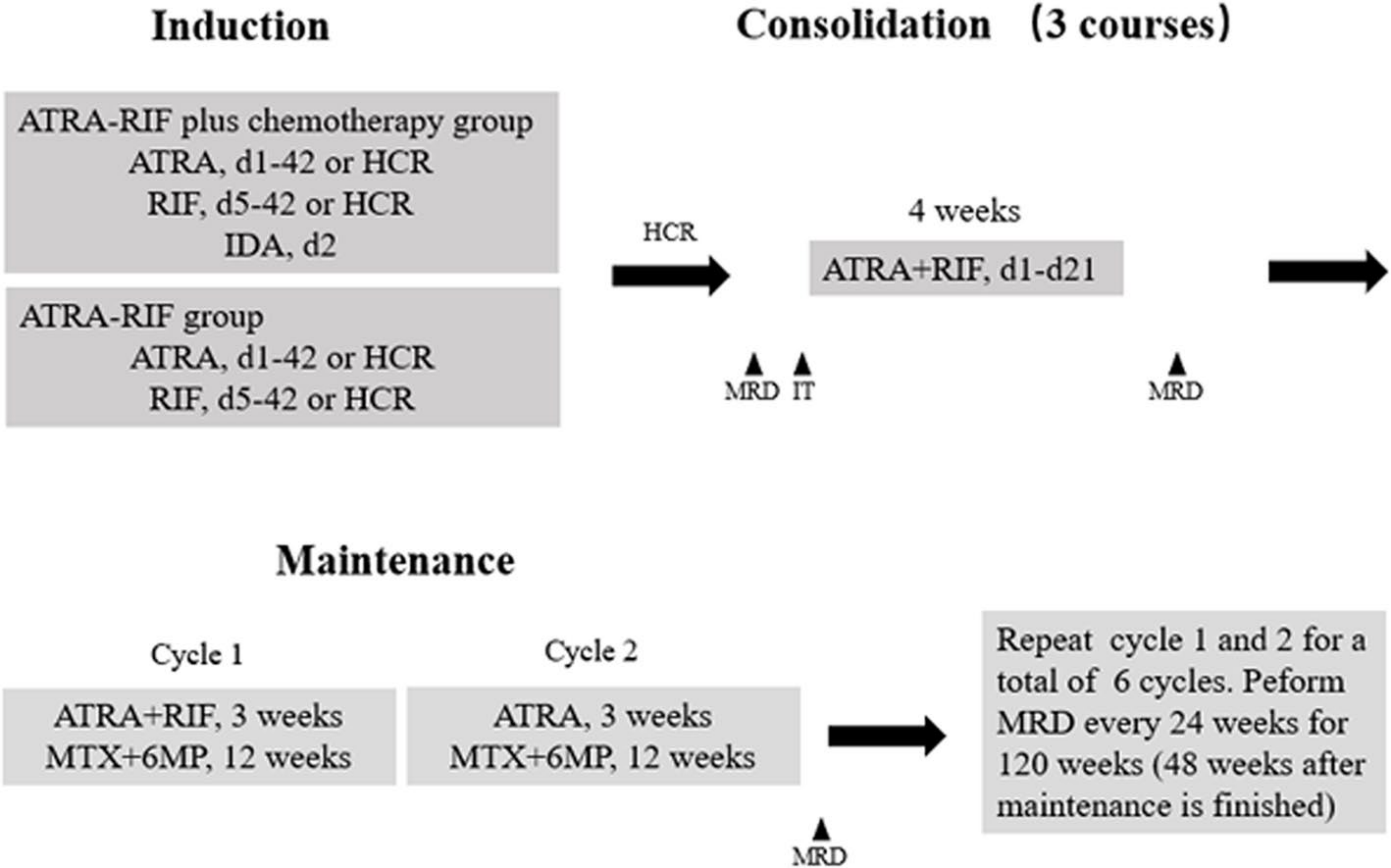
Treatment groups. HCR, hematological complete remission; MRD, minimal residual disease; IT, intrathecal injections; 6MP, 6-Mercaptopurine

Children with NHL APL will be randomly assigned by a computer-generated, random-allocation schedule to one of two groups: ATRA-RIF plus chemotherapy group (experimental group) and ATRA-RIF group (control group) (Figs. 2 and 3).

### Induction therapy

Induction therapy contains ATRA and RIF, and addition of one dose of idarubicin (IDA) for patients in experimental group. ATRA will be administrated once the diagnosis of APL is suspected based on morphological features. RIF will be given when the diagnosis of APL is genetically confirmed (5–6 days later from the day of morphological diagnosis).

The control group will receive oral ATRA at 25 mg/m^2^/day from day 1 until day 42 or HCR and oral RIF at 0.135 g/kg/day from day 5 until day 42 or HCR.

The experimental group will receive the same treatment as those assigned to the control group, with addition of IDA at 10 mg/m^2^ administrated on day 2.

Adverse events during induction treatment should be monitored mainly including coagulopathy, leukocytosis, DS, and QT prolongation. Blood routine and/or coagulation test should be performed every day or twice a day if there are leukocytosis and/or coagulopathy. Liver and kidney function tests and electrocardiogram should be performed every week.

At the initiation of and during induction therapy, when patients’ WBC count was over 10 × 10^9^/L, hydroxyurea (100 mg/kg/day) was administered until WBC < 10 × 10^9^/L. Dexamethasone (0.3 mg/kg/day) was given if differentiation syndrome or ATRA-associated pseudotumor cerebri was suspected. The use of heparin or low-molecular weight heparins for management of coagulopathy was not mandatory but encouraged because ATRA might increase thrombosis risk [20–22]. Heparin was given at a low dose of 0.5–1 mg/kg intravenously for 24 h daily if indicated until the coagulopathy resolved.

Transfusions of platelet and fresh-frozen plasma, cryoprecipitate, and/or human fibrinogen were given for the aims of maintaining platelet counts greater than 30 × 10^9^/L and fibrinogen greater than 1.5 g/L, respectively.

### Consolidation therapy

Consolidation will start when patients achieve HCR. Patients receive three courses of consolidation therapy every 4 weeks. Each consolidation consists of ATRA (25 mg/m^2^/day, d1-d21) and RIF (0.135 g/kg/day, d1-d21). Intrathecal injections (IT) are administered on day 1 of each course (Table 1). Blood routine, liver and kidney function tests, and electrocardiogram should be performed before the beginning of each consolidation.

**Table 1:** Drug dose for intrathecal injection

Age	Ara-C	DXM
0–1	15 mg	2 mg
1–3	20 mg	2 mg
> 3	20 mg	4 mg

### Maintenance therapy

Cycle 1: ATRA (25 mg/m^2^/day, weeks 1–3), RIF (0.135 g/kg/day, weeks 1–3), MTX (20 mg/m^2^/week, weeks 1–12), 6-mercaptopurine (6MP, 50 mg/m^2^/day, weeks 1–12).

Cycle 2: ATRA (25 mg/m^2^/day, weeks 1–3), MTX (20 mg/m^2^/week, weeks 1–12), 6MP (50 mg/m^2^/day, weeks 1–12).

Cycles 1 and 2 are repeated for a total of 6 cycles.

### Relevant concomitant care permitted or prohibited during the trial

Participants will continue to receive standard care and treatment from a specialized physician throughout the study. There are no limitations on appropriate concurrent care for patients, who are also permitted to sustain their routine healthcare regimen.

### Baseline characteristics

Baseline characteristics in each group will be analyzed using descriptive statistics, including means or medians for continuous variables and percentages for categorical variables.

### Events

Every case of hyperleukocytosis, DS, coagulopathy events, infection, gastrointestinal reaction, pseudotumor cerebri, and treatment-related toxicity of the liver, kidney, and heart occurring during the study must be recorded. The following information will be recorded: occurrence time, severity, duration, adopted measure, and the outcome of the adverse event.

Any serious adverse events occurring during the study period should be documented. If the serious events occur, investigators should determine whether participants should be withdrawn from the study depending on the patient’s condition and must take the necessary steps immediately and report to the ethics committee. Serious adverse events that are still ongoing at the end of the study must be followed up to determine the final outcome. Additionally, participants have the autonomy to discontinue their involvement in the trial at any point and for any reason. Comprehensive explanations for the withdrawal of each participant will be provided in the study results.

### Follow-up monitoring

Minimal residual disease (MRD) by qRT-PCR will be monitored at following treatment points: at the end of induction and consolidation using bone marrow samples and then every 24 weeks thereafter using blood or bone marrow samples for 120 weeks (48 weeks after maintenance is finished) [23]. If MRD is positive (PML-RARa ≥ 0.01% by qRT-PCR) during or after maintenance therapy, patients will be considered as having molecular relapse of APL, and qRT-PCR will be repeated after 1–2 weeks for confirming the relapse. Hematologic relapse is defined by bone marrow blasts of 5%, reappearance of blasts in the blood, or development of extramedullary disease.

### Study end points

The primary endpoint is the incidence of hyperleukocytosis, assessed at the end of the induction treatment. The secondary endpoints include the incidence of DS, hospital days of induction, time from diagnosis to HCR, the rate of molecular complete remission (MCR) at end of induction treatment, and the consumption of blood components including the amount of platelets, plasma, and cryoprecipitate used during induction.

### Data collection and management

We will inform patients about the importance of follow-up for health monitoring and early detection of relapse and will maintain regular telephone contact with patients to promote participant retention and complete follow-up. Patient information will be entered into the paper case report forms (CRFs) promptly and synchronously with input into the electronic CRF. Researchers must ensure that the data is true, complete, and accurate. After completing the observation of study cases, the data will be entered into the electronic data record as soon as possible, with entry performed by authorized personnel into the database. We will use the latest version of the Medical Dictionary for Regulatory Activities (MedDRA) to code adverse events (AEs) and medical history and the latest version of the World Health Organization Drug Dictionary (WHODRUG) to code concomitant medications. Additionally, researchers must ensure the privacy rights of patients participating in the clinical trial (anonymity). In all submitted documents, patients’ identities can only be identified using clinical trial identification codes (such as hospitalization numbers). Researchers must also properly safeguard the original documents containing personal information, such as the names and addresses of clinical trial participants. Any unexpected problems during this process should be documented and promptly reported. Furthermore, we will collect demographic and disease characteristics, including sex, age, height, weight, and medical history, to determine whether the two randomized groups are similar.

The ethics committee will oversee the researchers and all facets of the study to ensure strict adherence to ethical principles and the safeguarding of patient health and dignity. Should any ethical breaches occur, corrective measures will be implemented, and the study may be halted if necessary. The committee will monitor the integrity and accuracy of data collection to control its quality. The committee will also decide whether member hospitals would be included in the final analysis based on the authenticity of the data they submit. Any modifications to the study protocol and relevant documents must be submitted to the ethics committee for review and can only be implemented after obtaining the committee’s approval. The serious adverse event (SAE) and suspected unexpected serious adverse reaction (SUSAR) reports, as well as protocol violation reports, must be submitted to the ethics committee in a timely manner. Specifically, SAEs at this center must be reported within 24 h, while SAEs at other centers must be reported at least every 3 months; SUSARs must be reported at least every 6 months. Additionally, protocol violations must be reported to the ethics committee within 1 month of discovery. Annual reports should be submitted every year, and if the clinical study is suspended or terminated early, a discontinuation report must be submitted.

Data sharing is not applicable to this protocol paper as no data sets have been generated or analyzed in current. The results should be made public within 24 months of the end of the study. The end of the study is the time point at which the last data item is to be reported, or after the outcomes of data are sufficiently mature for analysis. A full report of the outcomes should be made public no later than 3 years after the end of the study. Confidential information pertaining to participants will remain undisclosed in all publications. Results will also be available through Chinese Clinical Trials Registry.

### Statistical analysis

The analysis populations for this study are defined as follows: the Full Analysis Set (FAS) includes all participants who signed informed consent and will be used for baseline analysis. The Safety Analysis Set comprises all participants who received the SCCCG-APL 2020 protocol and had at least one safety assessment after induction, while the Per-Protocol Analysis Set (PPAS) includes participants who fully adhered to the treatment regimen of either the experimental or control group without any validated protocol deviations and who completed the induction treatment along with minimal residual disease (MRD) testing. Additionally, for the population with protocol deviations, we will conduct an intention-to-treat (ITT) analysis, and multiple imputation will be applied to handle missing data.

Interim analysis is not applicable to this study. Firstly, the planned sample size is adequate to ensure robust statistical power at the conclusion of the study. Interim analyses might not capture the full spectrum of treatment effects, which could lead to premature conclusions and potentially inflate type I error rates. Furthermore, conducting interim analyses could introduce biases, as they may influence the treatment allocation or participants’ behaviors. We wanted to maintain the integrity of the study results without external influences. Additionally, previous study has already demonstrated the safety of anthracycline. Given the nature of our study and the regulatory framework, we believe that a final analysis at the end of the study will provide a clearer and more comprehensive understanding of the treatment’s efficacy and safety.

Statistical analysis will be performed using the SPSS software version 20.0. Descriptive analysis (calculations of averages, frequencies, proportions, or rates) will be conducted. Comparisons will be made using Student’s *t* test or the Mann–Whitney *U* test for comparison of continuous variables and Pearson’s *χ*^2^ test for dichotomous variables. Student’s *t*-test will be used to compare normally distributed variables, while Mann–Whitney *U* test will be used to compare variables with non-normal distribution. The chi-square test or Fisher’s exact test will be used to compare categorical variables between the two groups. Survival functions will be estimated using the Kaplan–Meier method and will be compared using the log-rank test. All statistical tests will be two-sided, and *P* < 0.05 is statistically significant.

### Standard Protocol Items: Recommendations for Interventional Trials (SPIRIT)

This protocol has been written in accordance with the SPIRIT guidelines [24]. The SPIRIT checklist is available in Supplementary File 1.

## Discussion

To our knowledge, SCCCG-APL is the first multicenter randomized clinical trial to compare the safety, efficacy, and feasibility between induction therapies with and without addition of low dose chemotherapy to ATRA-arsenic combination in APL. DS is one of the major causes of early death in APL [11]. Circumstantial evidence deriving from different clinical reports suggests that the incidences of leukocytosis and DS during induction is higher in pediatric than in adult patients and also higher in pediatric patients receiving the ATRA-arsenic combination as induction therapy than in those receiving addition of low intense chemotherapy to the combination [1, 3]. Therefore, this study is particular important for pediatric patients.

Our previous study (SCCLG-APL) showed that oral RIF is at least as effective and safe as intravenous ATO for the treatment of pediatric APL, with the advantage of reducing hospital stay [1, 25]. Moreover, the SCCLG-APL study also found that the arsenic concentrations of arsenic were not significantly different between the children treated with RIF at 135 mg/kg/day and ATO at 0.16 mg/kg/day, which were 0.5 µmol/L to 1 µmol/L in most patients, indicating that the dose of RIF at 135 mg/kg/day may be an appropriate therapeutic dose for pediatric patients [26]. Therefore, ATO at 0.16 mg/kg/day is replaced with RIF at 135 mg/kg/day in the ongoing SCCCG-APL study.

Beside DS, coagulopathy including hemorrhage and thrombosis triggered by APL blasts is another important adverse event that occurs before HCR of APL. There have also been circumstantial evidences suggesting that median time to achieve HCR could be shorter in pediatric patients if receiving addition of low intense chemotherapy to the ATRA-arsenic combination in induction therapy [1, 3]. Another interesting question is thus raised whether an addition of low intense chemotherapy will be more effective in induction in terms of shortening the time to reach HCR as well as increasing the rate of MCR at the end of induction.

In the ongoing SCCCG-APL study, the cumulative doses of anthracycline will be very low. The experimental group receive only one dose of IDA at 10 mg/m^2^ (in terms of doxorubicin, 50 mg/m^2^/day [Children’s Oncology Group. Long-term follow-up guidelines for survivors of childhood, adolescent, and young adult cancers, Version 6.0- October 2023. Available from: http://www.survivorshipguidelines.org/]) on the day 2 of induction.

Although survival rate of APL approaches 95% following contemporary treatment, the issue of how best to treat patients with APL remains to be answered. Literature reports on children with APL are limited. Many treatment strategies for the treatment of pediatric APL have been based on those for the treatment of adult patients. However, there are important distinctions between adult and pediatric APL [19], and treatment strategies proved successful in adults need to be verified in pediatric patients. The SCCCG-APL study mainly focus on the safety, efficacy, and feasibility of the different induction treatment strategies in pediatric patients and hopefully reach a conclusion on whether low intense chemotherapy is necessary for non-high-risk pediatric APL.

### Trial status

The protocol is version 1.0, date August 01, 2020. Recruitment began in October 2020 and scheduled to end in October 2024.

## Supplementary Information

Supplementary Table 1. 29 hospitals in South China enrolled in SCCCG-APL study

Supplementary File 1. SPIRIT checklist

## Data Availability

All data and materials can be obtained from the corresponding author.

## Abbreviations

ATRA: All-trans retinoic acid
APL: Acute promyelocytic leukemia
DS: Differentiation syndrome
ATO: Arsenic trioxide
RIF: Realgar-Indigo naturalis formula
NHR: Non-high risk
HCR: Hematological complete remission
SCCCG: South China Children Cancer Group
IDA: Idarubicin
MRD: Minimal residual disease
CRFs: Case report forms
