# Supplementary material for: Induction treatments with and without addition of one dose anthracycline to all-trans retinoid acid and arsenic in pediatric non-high-risk acute promyelocytic leukemia: study protocol for a randomized controlled trial
Source: Trials. 2024 Dec 18;25:819. doi: 10.1186/s13063-024-08664-y (PMC11654196; doi:10.1186/s13063-024-08664-y)
Supplement: Supplementary file 1 — Supplementary Table 1. 29 hospitals in South China enrolled in SCCCG-APL study [file 13063_2024_8664_MOESM1_ESM.docx]

Supplementary Table 1. 29 hospitals in South China enrolled in SCCCG-APL study

| NO. | Hospital |
| --- | --- |
| 1 | First Affiliated Hospital, Sun Yat-Sen University |
| 2 | Sun Yat-Sen Memorial Hospital, Sun Yat-Sen University |
| 3 | Third Affiliated Hospital, Sun Yat-Sen University |
| 4 | Guangzhou First People's Hospital |
| 5 | The First Affiliated Hospital of Guangdong Medical University |
| 6 | Shunde Women's and Children's Hospital of Guangdong Medical University |
| 7 | The First Affiliated Hospital of Guangzhou Medical University |
| 8 | Zhongshan People's Hospital |
| 9 | Second Xiangya Hospital, Central South University |
| 10 | Jiangxi Province Children's Hospital |
| 11 | The First Affiliated Hospital of Shantou University |
| 12 | The Second Affiliated Hospital of Shantou University |
| 13 | The First Affiliated Hospital of Nanchang University |
| 14 | Liuzhou People's Hospital |
| 15 | Hainan Province People's Hospital |
| 16 | Huizhou Central People’s Hospital, Huizhou |
| 17 | First People’s Hospital of Huizhou, Huizhou |
| 18 | Shenzhen Children’s Hospital |
| 19 | Fujian Medical University Union Hospital |
| 20 | First Affiliated Hospital of Xiamen University |
| 21 | Xiangya Hospital, Central South University |
| 22 | Zhujiang Hospital, Southern Medical University |
| 23 | Zhuhai Maternal and Child Health Care Hospital |
| 24 | Fifth Affiliated Hospital, Sun Yat-Sen University |
| 25 | Hunan People’s Hospital |
| 26 | The Affiliated Hospital of Guizhou Medical University |
| 27 | Dongguan Taixin Hospital |
| 28 | Nanfang hospital, Southern Medical University |
| 29 | Foshan Maternal and Child Health Care Hospital |
